# Validity Evidence of the TRIACOG-Online Administered In-Person to Adults Post Stroke

**DOI:** 10.3390/brainsci15070737

**Published:** 2025-07-10

**Authors:** Luana Comito Muner, Guilherme Domingos Martins, Ana Beatriz Santos Honda, Natália Becker, Jaqueline de Carvalho Rodrigues

**Affiliations:** 1 Postgraduate Program in Human Development Sciences, Universidade Presbiteriana Mackenzie, Sao Paulo 01302-907, SP, Brazil; luanamuner@gmail.com (L.C.M.); abeatriz.sh@gmail.com (A.B.S.H.); 2 Departamento de Psicologia, Pontifícia Universidade Católica do Rio de Janeiro, Rio de Janeiro 22451-900, RJ, Brazil; domingos.mguilherme@gmail.com

**Keywords:** neuropsychological assessment, validity evidence, computerized testing

## Abstract

**Background/Objectives**: Neuropsychological assessment tools adapted for digital formats are crucial to expanding access and improving cognitive evaluation in post-stroke patients. This study aimed to examine the reliability, convergent validity, and criterion-related validity (concurrent and known-groups) of TRIACOG-Online, a computerized cognitive screening tool designed to assess multiple domains in post-stroke adults in person or remotely. **Methods**: 98 participants (47 neurologically healthy adults and 51 post-stroke patients) completed a sociodemographic questionnaire, the Mini-Mental State Examination—MMSE, G-38—Nonverbal Intelligence Test, and the TRIACOG-Online assessment. Evaluations were conducted in person, computer mediated. **Results**: TRIACOG-Online demonstrated high internal consistency (Cronbach’s α = 0.872; McDonald’s ω = 0.923). Statistically significant differences were found between groups in episodic memory, attention, executive functions, and numerical processing, with healthy individuals outperforming post-stroke participants. Effect sizes were medium to large in several domains, especially for visual memory. Validity evidence based on the relationship with external variables was supported by negative correlations with age and positive correlations with education and reading and writing habits, particularly in the clinical group. Educational level showed stronger associations with verbal memory and language, suggesting a protective role in post-stroke cognitive performance. TRIACOG-Online scores demonstrated evidence of convergent validity with MMSE and G-38. **Conclusions**: TRIACOG-Online shows strong psychometric properties for the cognitive assessment of post-stroke adults. Its computerized format represents a promising tool for clinical and research use in neuropsychology, especially for bedside applications.

## 1. Introduction

Stroke is a sudden event that occurs abruptly in the cerebrovascular system and results in a neurological deficit that persists for at least 24 h in the patient [1]. It has a high global incidence and is the second leading cause of death worldwide [2]. Beyond its acute impact, stroke can lead to specific impairments, including dysphagia, aphasia, visual problems, neuropathic pain, cognitive impairments, and mood disorders, which may become permanent and compromise patients’ quality of life and functional independence [3]. It is estimated that over 100 million people live with its consequences throughout their lives [2].

A key strategy for evaluating neuropsychological functions, starting in the acute phase of stroke, involves the use of cognitive screening instruments or mental status assessments [4]. Screening instruments such as the Mini-Mental State Examination (MMSE) and the Montreal Cognitive Assessment (MoCA) are commonly used in post-stroke evaluations, although they were originally designed to assess patients with dementia, particularly Alzheimer’s disease, as they prioritize tasks related to orientation and memory. However, these functions are not primarily impaired in post-stroke patients, which may lead to underdiagnosis of cognitive deficits in this population. Furthermore, these instruments include relatively few items targeting language, executive functions, and processing speed—domains frequently impaired in this population [5]. More recently, the Oxford Cognitive Screening (OCS) was developed to avoid the confounding effects of aphasia and neglect that are frequent in stroke patients, and it covers five core cognitive domains (attention, language, memory, number, and praxis) [6]. Demeyere et al. [7] found higher sensitivity for the OCS than the MoCA in detecting cognitive impairments in stroke patients (88% vs. 79%), although it has been adopted in different countries, such as the Netherlands and Italy [8], they are countries with higher educational level and differences considering cultural and language aspects. In this context, the Cognitive Screening (TRIACOG) was developed in Brazil to address the need for sensitive, low-cost and quickly tools to assess neuropsychological functions in poststroke adults [9,10]. TRIACOG comprises 22 tasks designed to assess eight core neuropsychological domains: attention, executive functions, orientation, language, verbal and visual episodic memory, praxis, processing speed, and numerical processing, in approximately 20 min [11]. Norms, cut-off scores, and interpretative guidelines are available in the instrument manual [10]. Psychometric studies have demonstrated evidence of criterion validity—including concurrent, contrasting groups, and convergent validity—along with response process validity, inter-rater reliability, and test-retest reliability of its pencil-and-paper version [9,10,11,12].

The COVID-19 pandemic emphasized the need for remote neuropsychological assessments, especially for post-stroke patients. There is also growing demand for cognitive evaluations to reach individuals in remote regions or those living abroad without access to in-person services. In this context, TRIACOG was adapted into a computerized version—TRIACOG-Online [5]. This version includes a computerized application, online scoring protocol, presenting stimuli on a computer screen and responses registered by the evaluator throughout a tablet. TRIACOG-Online was originally designed for remote administration; however, it can also be administered in person, with computer mediation facilitating standardized data entry and reducing costs associated with paper-based protocols. Muner et al. [5] led the adaptation process, which included expert review, and a pilot study. Experts evaluated content validity, the adequacy of digital formats, and item clarity, confirming that TRIACOG-Online preserved the theoretical structure and cognitive domains of the original version. The pilot study, conducted with stroke patients and healthy adults, demonstrated feasibility, clarity of instructions, and usability of the interface in both in-person and remote formats, supporting its readiness for broader psychometric evaluation.

To expand research on psychometric properties of the TRIACOG-Online, this study represents the second stage in the validation of TRIACOG-Online, focusing on the psychometric evaluation of its computerized format in a supervised, in-person context. Although the tool was developed for both in-person and remote administration, this initial phase aimed to verify the feasibility and integrity of the adapted tasks before remote implementation. Specifically, we aimed to (a) investigate reliability through internal consistency analysis; (b) assess criterion validity based on comparisons between healthy adults and post-stroke patients; (c) examine validity evidence based on the relationship with external variables (age and education); (d) examine convergent validity; and (e) verify its feasibility in an in-person, examiner-mediated setting, as a preliminary step before remote administration testing with stroke patients. Accordingly, we expect (a) high internal consistency (Cronbach’s Alpha and McDonald’s omega > 0.75); (b) higher TRIACOG-Online scores among healthy adults compared to post-stroke individuals, controlling for education and with moderate effect sizes (>0.10); (c) negative correlations between age and TRIACOG-Online, and positive correlations with years of education and TRIACOG-Online; and (d) positive correlations with cognitive screening instruments, in line with findings from the pencil-and-paper version [9,10,11,12].

## 2. Materials and Methods

### 2.1. Participants

A total of 102 individuals participated in this study, divided into two groups: (1) a clinical post-stroke group and (2) a neurologically healthy control group (Table 1). The sample size needed to identify a difference of dz = 0.20, considering a probability of α = 0.05 and statistical power 1 − β = 0.80, is of 95 individuals. The software used for the sampling calculation was G*Power version 3.1.9.4 (Faul et al., 2007 [13]). The post-stroke group included 51 patients recruited from the stroke unit of a general hospital in northern Brazil. Participants ranged in age from 27 to 86 years, with a majority being female (*n* = 28; 54.90%). Regarding education, 11.76% were illiterate (*n* = 6), 35.29% had incomplete primary education (*n* = 18), 9.81% completed primary education (*n* = 5), 25.49% completed secondary education (*n* = 13), and 15.69% had higher education (*n* = 8). One participant did not report on educational background (1.96%), and years of education ranged from 0 to 19. Inclusion criteria for this group were the following: age over 18, Brazilian nationality, native speaker of Brazilian Portuguese, no other diagnosed neurological conditions, at least one stroke episode confirmed by neurological evaluation, preserved consciousness and alertness during assessment, and stroke occurrence between 24 h and seven days before evaluation (acute stage).

The healthy control group included 51 adults without any history of neurological or psychiatric conditions and no indication of neurocognitive disorder. Participants were recruited by convenience sampling based on availability and accessibility. Four individuals were excluded for not completing the TRIACOG-Online assessment, resulting in a final sample of 47 participants. In this group, ages ranged from 21 to 79 years, with most participants being female (*n* = 41; 87.23%). Educational background was distributed as follows: 14.89% with incomplete primary education (*n* = 7), 2.13% with completed primary education (*n* = 1), 25.53% with secondary education (*n* = 12), 48.94% with higher education (*n* = 23), and 8.51% with postgraduate education (*n* = 4). Years of education ranged from 3 to 25.

Both groups were recruited through convenience sampling, based on availability and accessibility of participants. While this approach was necessary due to the exploratory nature of the study and the clinical constraints associated with recruiting hospitalized stroke patients, it may have introduced sample bias and limited the generalizability of findings. These limitations are further discussed in the final section of the manuscript.

A statistically significant difference was observed in the distribution of sex between groups, with a predominance of females in the healthy group. No significant differences were found in age. Educational levels were higher in the healthy group. Most participants in both groups were right-handed. In the clinical group, all individuals had experienced at least one stroke, most of which were ischemic and located in the cortical region, particularly in the frontal and temporal lobes (see Table 1). None of the participants underwent surgical intervention due to stroke.

### 2.2. Instruments

All instruments were administered individually and in person, using a computerized version of the protocol developed via Google Forms. The assessment was conducted on a tablet and a screen of at least 10 inches with internet access. The evaluator guided participants verbally and recorded their responses in the online form.

Participants first provided consent through a digital informed consent form. They then completed a questionnaire addressing health conditions, habits, and sociodemographic variables. In addition, the clinical group answered questions related to stroke characteristics, including lesion site, time since stroke, and results of neurological assessments. Boths groups were assessed with the Mini-Mental State Examination—MMSE [14], a cognitive screening tool composed of 20 items assessing orientation (spatial and temporal), memory, attention, language, reading, and writing. The Brazilian version used in this study follows the Brazilian adaptation, with supporting psychometric evidence [15,16]. The subtests include temporal orientation (5 points), spatial orientation (5 points), repetition (3 points), calculation (5 points), recall (3 points), and language (9 points), yielding a maximum score of 30 points. They were also assessed with the G-38—Nonverbal Intelligence Test [17], a computerized nonverbal intelligence test designed to assess the general intelligence factor (g). It consists of one example item followed by 38 items grouped into six categories according to the type of problem-solving required. Each item presents a stimulus matrix, and the participant must identify the correct completion by selecting one of six response options. The current version was published in 2018, and its validity and reliability have been demonstrated in Brazil [17]. The analyses were based on the total number of correct responses, ranging from 0 to 38 points.

Finally, all participants completed the TRIACOG-Online assessment [5,10], with stimuli and scoring protocol delivered via computer. The instrument assessed the following domains, in approximately 20 min: temporal orientation (2 points); immediate and delayed verbal episodic memory (6 points each); constructive praxis (figure copy—24 points; clock drawing—9 points) and ideomotor praxis (1 point); visual episodic memory (24 points); attention/working memory (10 points); executive functions (phonemic verbal fluency—word count, inhibition and switching; rapid automatized naming—24 items); processing speed (time); language (naming—4 points; oral comprehension—2 points; writing—1 point; vocabulary—2 points; reading—14 points; inferential processing—2 points; repetition—4 points; dictation—4 points); and numerical processing (7 points). Tasks such as figure copy, naming, oral comprehension, and reading were completed directly on the tablet screen. Paper and pencil were used for tasks such as constructive praxis, visual memory reproduction, dictation, and numerical calculations. The computerized version of TRIACOG demonstrated a Cronbach’s alpha of 0.87. Although basic computer literacy was not formally assessed, all participants received standardized instructions and were supported by the evaluator during the digital tasks. Still, we acknowledge that limited digital experience—especially among older or less educated participants—may have influenced performance in specific domains involving direct interaction with the digital interface.

### 2.3. Data Collection

Assessments lasted approximately 60 min and were conducted by trained evaluators (including all the instruments). Clinical participants were assessed at bedside during hospitalization. Healthy participants were recruited through online advertisements and evaluated at a university clinic. All assessments took place in quiet, well-lit rooms without interruptions.

### 2.4. Data Analysis

Normality of TRIACOG-online subtest scores was assessed using the Shapiro–Wilk test, which indicated non-normal distributions. Therefore, nonparametric analyses were applied. Internal consistency was measured using Cronbach’s alpha, with interpretation thresholds as follows: very low (α ≤ 0.30), low (0.30 < α ≤ 0.60), moderate (0.60 < α ≤ 0.75), high (0.75 < α ≤ 0.90), and very high (α > 0.90). McDonald’s omega was also calculated, with acceptable values ranging between 0.70 and 0.95. Criterion validity (known-groups validity) was examined using ANCOVA, controlling for educational level. Effect sizes were calculated using eta-squared (η^2^), interpreted as small (<0.01), medium (0.02–0.06), or large (>0.14). A post hoc power analysis was conducted using G*Power 3.1 for the ANCOVA model (two groups, one covariate). With a total sample of N = 98, an observed effect size of f = 0.2, and α = 0.05, the achieved power was approximately 0.50.

Validity evidence based on the relationship with external variables was assessed using Spearman’s correlations between TRIACOG-Online scores and participants’ age, years of education, and frequency of reading and writing habits. Convergent validity was performed with Spearman’s correlations between TRIACOG-Online scores and MMSE and G-38. All analyses were performed using Jeffrey’s Amazing Statistics Program [18].

### 2.5. Ethical Considerations

This multicenter study was conducted by partner universities and approved by the respective institutional review boards. All participants were recruited from Roraima, Brazil.

## 3. Results

### 3.1. Reliability and Internal Consistency

Cronbach’s alpha coefficients for the TRIACOG-Online subtests ranged from α = 0.85 to α = 0.87, indicating high internal consistency within the ideal range for psychological instruments [19]. McDonald’s omega values ranged from ω = 0.92 to 0.93. Table 2 presents coefficients for each subtest.

### 3.2. Criterion Validity Based on Group Comparisons

Before conducting the ANCOVA, assumptions of normality, homogeneity of variances (Levene’s test), and homogeneity of regression slopes were tested and met. No imputation procedures were necessary, as there was no missing data for the variables included in the analysis, except for a single participant with missing educational information. Table 3 presents the means and standard deviations of TRIACOG-Online scores for both the healthy and stroke groups, along with ANCOVA results (controlling for years of education). Statistically significant differences were found between groups in the following domains: verbal episodic–semantic memory (total score), constructional praxis—figure copy, auditory attention/working memory (total score and attention span), verbal fluency, executive functions (rapid automatized naming—correct responses), reading, numerical processing, and visual episodic memory. In all cases, the healthy group outperformed the stroke group. Effect sizes (η^2^) were medium for nine domains and large for visual memory. No differences were found between groups in tasks of orientation, verbal memory—immediate recall, ideomotor praxis, processing speed, oral and written comprehension, naming, vocabulary, inference processing, repetition, and dictated writing. However, the post-stroke group presented lower scores in these tasks, compared to the group of healthy adults (Table 3).

### 3.3. Validity Evidence Based on the Relationship with External Variables (Sociodemographic Variables)

Table 4 presents Spearman correlation coefficients between TRIACOG-Online subtest scores and age, years of education, and reading/writing habits, for each group separately. In both groups, age showed statistically significant negative correlations with TRIACOG-Online performance, ranging from weak to moderate magnitudes (r = −0.204 to r = −0.570; *p* < 0.01). In other words, higher age was associated with lower cognitive performance in verbal episodic–semantic memory, executive functions, reading, and visual memory.

Educational level was positively associated with a greater number of subtests for stroke patients (r = 0.373 to r = 0.600; *p* < 0.01), as visual and verbal episodic–semantic memory, constructional praxis, executive functions, language, and numerical processing. In the healthy group, education correlated weakly only with the time of the rapid automatized naming task. These positive associations indicate that higher education is linked to better cognitive performance, particularly in the post-stroke group.

Reading and writing habits were also positively correlated with performance across several TRIACOG-Online domains in both groups (r = 0.201 to r = 0.577; *p* < 0.01). Thus, more frequent engagement in reading and writing activities was associated with better cognitive outcomes in visual and verbal episodic–semantic memory, constructional praxis, auditory attention/working memory, executive functions, language, and numerical processing, both in healthy individuals and in those in the acute stage after stroke.

### 3.4. Convergent Validity Evidence Based on Related Constructs Assessed with Established Instruments

Convergent validity of the TRIACOG-Online was assessed using two established instruments: the MMSE and the G-38. First, we examined whether healthy controls and post-stroke participants showed expected differences in performance on these external cognitive measures. Independent samples Welch’s *t*-tests revealed statistically significant group differences for both instruments. On the MMSE, the healthy participants (*M* = 25.61, *SD* = 1.26) outperformed stroke group (*M* = 23.85, *SD* = 2.95), *t*(64.64) = 3.74, *p* < 0.001, *d* = 0.77. Similarly, for the G-38, healthy participants (*M* = 14.71, *SD* = 8.15) outperformed the stroke group (*M* = 9.84, *SD* = 5.84), *t*(74.23) = 3.17, *p* = 0.002, *d* = 0.69. These findings support the criterion-related validity of both instruments and confirm expected cognitive disparities between clinical and non-clinical groups.

Next, Spearman correlations were calculated between TRIACOG-Online scores and total scores from the MMSE and G-38. These results are presented in Table 5. Statistically significant positive correlations of low to moderate magnitude (*r* = 0.20 to 0.68, *p* < 0.05) were found between most TRIACOG-Online subtests and the MMSE across both groups. A notable negative correlation was observed between MMSE scores and the number of errors on the Rapid Automatized Naming task (Healthy: *r* = −0.404, *p* < 0.001; Stroke: *r* = −0.497, *p* < 0.05), indicating that better global cognitive functioning was associated with fewer errors on this executive function task.

The pattern of correlations between conceptually similar subtests was also examined. For instance, TRIACOG’s orientation score correlated moderately with MMSE temporal orientation (*r* = 0.338 to 0.392). MMSE repetition, however, did not significantly correlate with TRIACOG’s immediate recall in either group, likely due to differences in task structure. MMSE calculation was negatively associated with TRIACOG’s numerical processing, but only in the control group, while MMSE recall was weakly correlated with TRIACOG’s delayed recall for controls. MMSE language scores were positively correlated with various TRIACOG language tasks, with exceptions for oral comprehension and dictation (both groups), and for vocabulary/semantic memory (stroke group).

Correlations between G-38 scores and TRIACOG-Online were generally positive and statistically significant, though typically lower in magnitude. These associations were more consistent in the control group than in the stroke group, supporting the convergent validity of the TRIACOG-Online and its sensitivity to cognitive differences, particularly in non-clinical populations.

## 4. Discussion

This study aimed to examine the reliability, validity, and feasibility of the TRIACOG-Online prototype for both healthy individuals and post-stroke patients, administered in person. It is important to note that, although TRIACOG-Online is designed also for remote use, this study focused on evaluating the psychometric properties of the adapted digital version in a face-to-face, digitally mediated environment. This was a necessary preliminary step to establish feasibility and preserve assessment integrity prior to remote administration testing. The analyses revealed adequate psychometric properties for the computerized version of the instrument, tested with the participant and examiner in the same physical setting. The results are discussed below by evidence.

### 4.1. Reliability and Internal Consistency

The findings from TRIACOG-Online subtests indicate that the instrument reliably measures the intended latent constructs. Internal consistency coefficients ranging from 0.70 to 0.95 are considered acceptable for establishing instrument reliability [3], and the values obtained in this study met those expectations. Aligned with our hypothesis, the instrument’s computerized version showed a high internal consistency, similar to the original pencil-and-paper version, indicating that it was adapted to preserve the assessment of the same constructs [5,10].

These results align with findings from other neuropsychological tools used in Brazil with post-stroke patients. For instance, the NEUPSILIN-L showed a Cronbach’s alpha of 0.93 and McDonald’s omega of 0.95 [20], while the MoCA showed good consistency (α = 0.74) in another study [21]. Additionally, previous studies found α = 0.78 for the MMSE and α = 0.81 for the MoCA [22].

Notably, TRIACOG-Online reliability was assessed for each cognitive domain, in contrast to tools like the MoCA and MMSE, which provide only the total score. As a result, interpretive differences must be considered. Moreover, consistency may vary across samples and contexts, emphasizing the need for cautious generalization. Still, the TRIACOG-Online demonstrates strong internal consistency, underscoring its reliability and precision.

### 4.2. Criterion Validity Based on Group Comparisons

Group comparisons using TRIACOG-Online replicated findings from the pencil-and-paper version reported [10,11,12], in which healthy individuals performed better in domains such as verbal episodic memory, auditory attention, working memory, verbal fluency, and rapid automatized naming. Also, in this study the patients demonstrated difficulty in tasks that assess functions usually impaired in post-stroke individuals: constructional praxis—figure copy, reading, numerical processing, and visual episodic memory.

These results were expected and are consistent with prior research indicating significant cognitive decline after stroke [11,23] thereby strengthening the criterion validity of TRIACOG-Online. One study [23] also reported persistent deficits one year after stroke, particularly in language, processing speed, and executive functions, though not in memory or MMSE performance, corroborating our results.

Additionally, healthy individuals tend to achieve higher scores on cognitive tasks, reinforcing the validity of TRIACOG-Online in distinguishing clinical from non-clinical groups [11,23]. Differences between the groups may reflect stroke-related cognitive impairments [24].

However, compared to the pencil-and-paper version, some results differed from our findings—the first reported more pronounced differences in some cognitive domains, such as orientation, clock drawing, processing speed, and numerical calculations [12], which were not observed in the present study. These discrepancies may be related to the mode of administration (in-person vs. remote) and sample characteristics. For example, prior studies with MoCA have shown that while results are generally comparable across formats, differences are more marked in domains like language and visuospatial skills [25]. These differences may be due to factors such as digital interface interaction and audio quality.

Regarding executive functions specifically, our findings are consistent with previous studies showing post-stroke impairments in domains such as working memory, verbal fluency, and rapid automatized naming. These tasks rely on mental flexibility, inhibition, and sustained attention—functions typically associated with frontal lobe activity, which is often compromised in stroke patients. In our sample, stroke survivors exhibited significantly lower scores in these executive domains when compared to healthy individuals. These results align with prior literature emphasizing executive dysfunction as a common post-stroke cognitive sequela [16,17]. Moreover, even in tasks where between-group differences did not reach statistical significance—such as verbal comprehension, naming, and inference processing—stroke patients still performed below controls, suggesting subtle deficits that may become more apparent in more demanding everyday cognitive activities. Furthermore, educational background is a relevant factor, as individuals with lower education levels tend to perform worse in executive and language domains [12].

### 4.3. Validity Evidence Based on the Relationship with External Variables

Our results showed a negative correlation between TRIACOG-Online performance and age, consistent with findings from other cognitive screening instruments and the original TRIACOG version [9,10,11,12]. Similar patterns were observed in studies using the MMSE [26] and MoCA [27]. These results confirm the validity evidence based on the relationship with external variables of TRIACOG-Online in capturing age-related cognitive decline, which is typically associated with reductions in memory, attention, processing speed, and executive functioning [28].

Although age-related decline is expected, it can be influenced by factors such as overall health, lifestyle, comorbidities, and age-related brain changes [29,30]. Educational attainment also correlated positively with TRIACOG-Online performance, particularly among stroke patients, echoing prior research [9,10,12,26,27].

While both groups were expected to show such correlations, significant associations with education were more evident in the post-stroke group, suggesting that education may act as a protective factor. These findings align with evidence that education positively influences domains such as episodic memory, processing speed, verbal fluency, and both crystallized and fluid intelligence [31].

Some studies have even suggested that education may have a greater effect on cognitive performance than age itself [32,33,34]. In our sample, this was particularly true in the post-stroke group, in which education was more strongly associated with verbal memory, language, and executive functioning. This supports the hypothesis that lower education levels may exacerbate age-related cognitive decline.

Reading and writing habits were also positively associated with TRIACOG-Online performance, supporting prior studies with the pencil-and-paper version [9,10,11,12]. The relevance of lifelong literacy habits has been demonstrated in both clinical and non-clinical populations [35], suggesting that maintaining such habits may help preserve and enhance cognitive functions regardless of health status. These findings further support the validity evidence based on the relationship with external variables of TRIACOG-Online, particularly in tasks involving memory and auditory attention, which are closely linked to literacy engagement in older adults [36].

### 4.4. Convergent Validity Evidence Based on Related Constructs Assessed with Established Instruments

The observed associations between TRIACOG-Online and the MMSE support findings from previous studies examining relationships between TRIACOG tasks and other screening instruments such as the MoCA-B [11]. These studies reported weak to moderate correlations, highlighting consistency in patterns across instruments. Similarly, a previous study [11] also found positive associations between TRIACOG and MoCA-B scores. Moreover, previous research has demonstrated moderate to strong correlations between cognitive screening tools such as the MMSE and MoCA [11,37,38].

Although these screening tools aim to measure the same underlying construct—general cognitive functioning—they differ in task formats and scoring procedures. For example, the MMSE does not incorporate timing in any of its tasks, which may explain the lack of correlation with time-based performance metrics in TRIACOG-Online. Despite these differences, the current findings provide additional support for the convergent validity of TRIACOG-Online and strengthen its utility as a valid tool for cognitive screening.

Convergent validity evidence was also supported by the low to moderate associations observed between TRIACOG-Online scores and the G-38, a general measure of intelligence. This is consistent with previous studies that found significant correlations between the Brain Assessment and MMSE [39], and correlations ranging from low to high between the MoCA and the Wechsler Adult Intelligence Scale—Third Edition (WAIS-III) in a neuropsychological assessment study [40]. A strong correlation between MMSE total scores and WAIS-III performance was also observed. Given the conceptual overlap between intelligence and broader cognitive functioning, the associations between TRIACOG-Online and the G-38 further support its convergent validity.

### 4.5. Limitations

This study has some limitations that must be addressed and taken into consideration when interpreting results. Firstly, convenience sampling was used, and participants were recruited from a single geographic region in Brazil, limiting the national representativeness and generalizability of the findings. The control group, for instance, was recruited based on accessibility, which may have contributed to demographic imbalances—particularly the higher proportion of women and participants with higher education. These characteristics may impact on the generalizability of our findings and should be addressed in future research using stratified or randomized sampling strategies. A post hoc power analysis indicated that the achieved statistical power for detecting group differences (ANCOVA, f = 0.2, α = 0.05) was approximately 0.50. Although this value is below the conventional threshold for adequate power (0.80), it reflects the exploratory nature of this preliminary study. The findings should therefore be interpreted as initial evidence of validity and feasibility, with future research needed to confirm these results in larger samples. Secondly, significant differences in education between groups suggest the need to stratify future samples by key variables such as age and education. We also acknowledge the sex imbalance between groups—particularly the predominance of women in the control group—which may have influenced the results. However, previous validation studies of TRIACOG and other neuropsychological instruments have not consistently identified sex differences in performance, reducing the potential bias introduced by this imbalance. Thirdly, this study did not include analyses of structural validity (e.g., factor analysis). This decision was made based on the limited sample size and the multidimensional design of TRIACOG-Online, which assesses distinct cognitive domains with a small number of items per domain. In such cases, factor analysis is not considered methodologically appropriate or conceptually meaningful. This is consistent with other validated screening tools such as the MMSE and MoCA, which also do not rely on factorial models in their original psychometric validation.

Additionally, stroke-related variables such as lesion site and time since stroke were not controlled and may have contributed to within-group variability. Finally, although TRIACOG-Online is designed for both in-person and remote use, this study only tested the instrument face-to-face, with both participant and examiner in the same setting. As such, our findings reflect the feasibility and validity of the computerized adaptation in a controlled environment.

Future studies should aim to expand the validity evidence of TRIACOG-Online through remote applications, comparing performance outcomes with those obtained in in-person assessments. Preliminary findings from ongoing teleneuropsychology research suggest that remote assessments often require the presence of a caregiver or facilitator familiar with technology. This individual typically assists with tasks such as navigating the interface, submitting images of written responses, and managing audio equipment (e.g., headphones and microphones). These practical limitations should be taken into account by clinicians planning to implement TRIACOG-Online or similar tools in remote neuropsychological evaluations.

## 5. Conclusions

This study examined the psychometric properties of TRIACOG-Online in healthy and post-stroke adults assessed in person, as a first step in the entire process of adapting an instrument to digital format, as recommended by the International Test Commission [41]. The results support the reliability of the instrument, evidenced by high internal consistency. Comparisons between groups demonstrated TRIACOG-Online criterion validity, with healthy individuals outperforming stroke patients on several cognitive tasks. The relationship between the results of TRIACOG-Online and other instruments reinforces its evidence of convergent validity.

Age and literacy habits were associated with TRIACOG-Online performance, providing evidence of validity based on the relationship with external variables. Although educational level was not consistently associated with performance across both groups, significant correlations were observed within the stroke group, suggesting that education may serve as a protective factor against the effects of cerebrovascular injury. Alternatively, the limited variability in educational levels within the healthy group may have reduced the strength of this relationship.

This study indicates that TRIACOG is a versatile tool for neuropsychological assessment in hospitalized post-stroke adults, available in both pencil-and-paper and computerized formats. The consistency of findings across studies highlights the instrument’s robustness and potential for use across diverse contexts and populations. TRIACOG-Online shows promise as a valuable resource for researchers and clinicians seeking to assess cognitive function in post-stroke populations. However, its applicability across broader contexts should be further investigated in future studies with diverse and representative samples.

## Figures and Tables

**Table 1:** Sociodemographic characteristics of the participants.

Parameters ^1^	Healthy (*n* = 47)	Stroke (*n* = 51)	F/χ^2^	*p*
Sex (M/F)	6/41	23/28	11.7	<0.001
Age *M* (*SD*)	54.2 (11.4)	56.6 (14.8)	55.6	0.211
Education *M* (*SD*)	12.9 (5.31)	8.54 (5.81)	26.5	0.002
Handedness (R/L)	48/1	49/0	1.01 ^2^	0.315
No. of strokes *M* (*SD*)	–	1.34 (0.68)	–	–
Stroke type (H/IN/IA/IT/AVI)	–	(2/34/5/0/1)	–	–
Lesion region (C/S/B)	–	(12/3/2)	–	–
Stroke location (F/T/O/P)	–	(7/7/3/1)	–	–

^1^ M = Male; F = Female; SD = Standard Deviation; R = Right; L = Left; H = Hemorrhagic; IN = Ischemic, not specified; IA = Atherothrombotic Ischemic; IT = Ischemic, thrombotic window; AVI = Ischemic Stroke; C = Cortical; S = Subcortical; B = Both; F = Frontal; T = Temporal; O = Occipital; P = Parietal. ^2^ Chi-square test (χ^2^).

**Table 2:** Cronbach’s α and McDonald’ ω coefficients for each subtest from TRIACOG-Online.

TRIACOG-Online Subtests	Cronbach’s α	McDonald’s ω
Orientation	0.872	0.921
Verbal episodic–semantic memory	0.862	0.918
Immediate recall	0.866	0.918
Delayed recall	0.868	0.922
Auditory attention/working memory	0.866	0.920
Forward span (auditory attention)	0.869	0.920
Backward span (working memory)	0.869	0.922
Visual memory	0.873	0.920
Figure copying	0.861	0.917
Clock drawing	0.862	0.921
Ideomotor praxis	0.872	0.923
Verbal fluency	0.865	0.921
RAN—correct answers	0.853	0.916
RAN—errors	0.855	0.917
RAN—time A	0.869	0.923
RAN—time B	0.868	0.922
RAN—time C	0.867	0.921
Oral comprehension	0.873	0.924
Naming	0.869	0.917
Vocabulary/semantic memory	0.872	0.921
Reading	0.856	0.917
Inferential processing	0.870	0.920
Written comprehension	0.872	0.921
Repetition	0.874	0.926
Writing (dictation)	0.872	0.925
Numerical processing	0.863	0.919

**Table 3:** Descriptive statistics, ANCOVA results, and effect sizes (η^2^) for TRIACOG-Online scores among healthy adults and post-stroke patients, controlling for years of education.

TRIACOG-Online Subtests	Healthy (*n* = 47)	Post-Stroke (*n* = 51)		ANCOVA					
	*M*	*SD*	*M*	*SD*	*df*	*MS*	*F*	*p*	*η* ^2^
Orientation	1.957	0.204	1.875	0.393	1	0.076	1.323	0.253	0.016
Verbal episodic–semantic memory	7.298	2.312	5.104	2.336	1	19.783	4.053	0.047 *	0.041
Immediate recall	4.702	0.998	3.688	1.446	1	1.43	1.079	0.302	0.012
Delayed recall	2.596	1.765	1.447	1.457	1	9.923	3.825	0.054	0.041
Constructional praxis									
Figure copying	21.106	4.574	13.771	9.859	1	436.674	9.346	0.003 **	0.092
Clock drawing	6.17	2.64	3.063	2.906	1	15.586	1.954	0.166	0.02
Ideomotor praxis	1.0	0.0	0.896	0.309	1	0.004	0.363	0.549	0.004
Auditory attention/working memory	8.255	1.811	6.771	2.166	1	19.692	5.088	0.027 *	0.053
Forward span (auditory attention)	4.894	0.598	4.188	1.142	1	3.778	4.802	0.031 *	0.048
Backward span (working memory)	3.362	1.538	2.583	1.528	1	6.22	2.733	0.102	0.031
Executive Functions									
Verbal fluency	5.467	2.17	3.565	2.247	1	26.253	7.168	0.009 **	0.075
RAN—correct answers	21.723	2.74	18.917	6.451	1	106.23	6.556	0.012 **	0.072
RAN—errors	1.822	2.434	4.723	6.412	1	31.927	2.071	0.154	0.024
Processing speed									
RAN—time A	5.894	1.914	5.479	2.917	1	0.055	0.011	0.917	1.362
RAN—time B	6.532	2.283	5.708	2.968	1	0.522	0.091	0.763	0.001
RAN—time C	7.043	2.265	6.063	2.999	1	9.599	1.827	0.180	0.022
Language									
Oral comprehension	0.979	0.146	0.939	0.242	1	0.008	0.222	0.638	0.003
Naming	3.979	0.146	3.563	0.92	1	0.272	3.434	0.068	0.038
Vocabulary/semantic memory	1.979	0.146	1.792	0.544	1	0.002	0.031	0.860	3.76
Reading	13.979	0.146	11.271	4.894	1	67.498	11.176	0.001 ***	0.108
Inferential processing	1.87	0.4	1.208	0.874	1	0.564	1.5	0.224	0.015
Written comprehension	0.957	0.204	0.875	0.334	1	0.151	2.291	0.134	0.027
Repetition	7.447	1.212	6.542	2.221	1	6.52	2.153	0.146	0.023
Writing (dictation)	2.66	1.403	2.146	1.571	1	1.421	0.686	0.410	0.008
Numerical processing	4.191	1.191	1.898	1.95	1	20.198	8.225	0.005 ***	0.065
Visual memory	16.644	6.516	8.646	9.005	1	827.494	15.687	0.001 ***	0.144

*M* = Mean; *SD* = Standard Deviation; *df* = degrees of freedom; *MS* = Mean Square; RAN = Rapid automatized naming. All tests were one-sided, with the specific hypothesis that the Healthy group would outperform the Stroke group. NaN = Variance in ideomotor praxis was equal to zero after grouping by condition. *p* values: *p* ≤ 0.05 (*), *p* ≤ 0.01 (**), *p* < 0.001 (***).

**Table 4:** Spearman correlations between TRIACOG-Online scores and sociodemographic variables (age, education, and reading/writing habits), by group.

TRIACOG-Online Subtests	Healthy (*n* = 47)			Post-Stroke (*n* = 51)		
	Age	Education	R/W Habits	Age	Education	R/W Habits
Orientation	−0.085	−0.01	0.136	−0.199	0.265	0.254
Verbal episodic–semantic memory	−0.328 ***	−0.111	0.435 ***	−0.367	0.566 ***	0.519
Immediate recall	−0.338 ***	−0.122	0.322 ***	−0.377 *	0.472 ***	0.321 *
Delayed recall	−0.256 *	−0.075	0.369 ***	−0.243	0.445 *	0.520 ***
Constructional praxis						
Figure copying	−0.297 *	−0.095	0.343 ***	−0.548 ***	0.532 ***	0.432 *
Clock drawing	−0.129	−0.199	0.364 ***	−0.393 *	0.437 *	0.354 *
Auditory attention/working memory	−0.127	0.022	0.397 ***	−0.183	0.373 *	0.434 *
Forward span (auditory attention)	−0.095	0.131	0.32 **	−0.085	0.425 *	0.338 *
Backward span (working memory)	−0.128	−0.055	0.326 **	−0.219	0.217	0.325
Executive Functions						
Verbal fluency	−0.205 *	−0.157	0.312 *	−0.324 *	0.600 ***	0.470 ***
RAN—correct answers	−0.395 ***	−0.186	0.465 ***	−0.516 ***	0.494 ***	0.403 *
RAN—errors	−0.466 ***	0.276	−0.359 ***	0.524 ***	−0.458 **	−0.325 *
Processing speed						
RAN—time A	0.019	0.262	0.099	−0.061	0.028	0.207
RAN—time B	0.081	0.292 *	0.201 *	−0.087	0.002	0.26
RAN—time C	−0.086	0.226	0.353 ***	−0.186	0.229	0.374 **
Ideomotor praxis	−0.036	0.155	0.218 *	0.012	0.051	0.242
Language						
Naming	−0.228 *	0.094	0.322 **	−0.26	0.475 ***	0.414 **
Oral comprehension	−0.102	−0.033	0.136	−0.208	0.169	0.130
Vocabulary/semantic memory	−0.074	−0.01	0.186	−0.006	0.219	0.212
Reading	−0.368 ***	−0.147	0.382 ***	−0.497 ***	0.586 ***	0.461 ***
Inferential processing	−0.204 *	−0.177	0.260 *	−0.230	0.488 ***	0.363 *
Writing (dictation)	−0.148	−0.102	0.037	−0.255	0.454 **	0.253
Repetition	−0.108	0.074	0.116	−0.156	0.001	0.043
Written comprehension	−0.162	0.038	0.267 *	−0.328 *	0.460 **	0.336 *
Numerical processing	−0.174	0.169	0.435 ***	−0.448 **	0.467 ***	0.444 **
Visual memory	−0.363 ***	−0.099	0.444 ***	−0.570 ***	0.573 ***	0.577 ***

R/W Habits = Reading and writing habits; RAN = Rapid automatized naming. *p* values: *p* ≤ 0.05 (*), *p* ≤ 0.01 (**), *p* < 0.001 (***).

**Table 5:** Spearman’s correlations between TRIACOG-Online scores and G-38, and MMSE.

TRIACOG-Online	Healthy (*n* = 47)		Stroke (*n* = 51)	
	MMSE	G-38	MMSE	G-38
Orientation	0.338 ***	0.060	0.392 **	0.169
Verbal episodic–semantic memory	0.437 ***	0.320 **	0.504 ***	0.212
Immediate recall	0.326 **	0.326 **	0.365 *	0.077
Delayed recall	0.358 ***	0.221 *	0.375 *	0.254
Constructional praxis				
Figure copying	0.461 ***	0.258 *	0.678 ***	0.319 *
Clock drawing	0.363 ***	0.269 *	0.576 ***	0.192
Auditory attention/working memory	0.294 **	0.180	0.246	−0.128
Forward span (auditory attention)	0.190	0.120	0.193	−0.033
Backward span (working memory)	0.273 **	0.151	0.176	−0.140
Executive Functions				
Verbal fluency	0.142	0.235 *	0.302 *	0.086
RAN—correct answers	0.375 ***	0.327 **	0.537 ***	0.167
RAN—errors	−0.404 ***	−0.323 **	−0.497 ***	−0.130
Processing speed				
RAN—time A	−0.052	−0.085	−0.034	−0.058
RAN—time B	0.045	−0.035	0.078	−0.018
RAN—time C	0.131	0.126	0.097	0.184
Ideomotor praxis	0.204 *	0.140	0.175	0.082
Language				
Naming	0.459 ***	0.317 **	0.535 ***	0.347 *
Oral comprehension	−0.132	0.041	−0.113	0.163
Vocabulary/semantic memory	0.266 **	0.201	0.274	0.178
Reading	0.447 ***	0.305 **	0.581 ***	0.246
Inferential processing	0.276 **	0.254 *	0.230	0.213
Writing (dictation)	−0.006	−0.014	0.141	0.244
Repetition	0.080	−0.047	0.170	−0.008
Written comprehension	0.224 *	0.090	0.434 **	0.195
Numerical processing	0.354 ***	0.355 ***	0.415 **	0.335 *
Visual memory	0.481 ***	0.297 **	0.602 ***	0.171

* = *p* ≤ 0.05, ** = *p* ≤ 0.01, *** = *p* < 0.001.

## Data Availability

The raw data supporting the conclusions of this article will be made available by the authors on request.
